# Supplementary material for: SyNDI: synchronous network data integration framework
Source: BMC Bioinformatics. 2018 Nov 6;19:403. doi: 10.1186/s12859-018-2426-5 (PMC6219086; doi:10.1186/s12859-018-2426-5)
Supplement: Supplementary file 5 — Files of the example presented in Additional file 4. This additional file is a zip package that contains all files used in Additional file 4. Detailed descriptions of each of these files are presented in a README file included in this zip package. (GZ 30411 kb) [file 12859_2018_2426_MOESM5_ESM.gz › SyNDITools/Meme2Fimo/meme_4.9.0_4.tar.gz!/meme_4.9.0/doc/mast.html]

MAST: Motif Alignment and Search Tool


# MAST: Motif Alignment and Search Tool

## Usage

mast <motif file> <sequence file> [options]

| Inputs | |
| <motif file> | file containing motifs to use; normally a MEME output file |
| <sequence file> | search sequences in FASTA-formatted database with motifs; |
| [-bfile <file>] | read background frequencies from <file> |
| [-dblist] | read the <sequence file> as a list of FASTA-formatted databases |
| Outputs | |
| [-o <dir>] | directory to output mast results; directory must not exist |
| [-oc <dir>] | directory to output mast results with overwriting allowed |
| [-hit\_list] | print a machine-readable list of all hits only; outputs to standard out and overrides -seqp |
| Which Motifs To Use | |
| [-remcorr] | remove highly correlated motifs from query |
| [-m <m>]+ | use only motif number <m> (overrides -mev); this can be repeated to select multiple motifs |
| [-c <count>] | only use the first <count> motifs or all motifs when <count> is zero (default: 0) |
| [-mev <mev>] | use only motifs with *E*-values less than <mev> |
| [-diag <diag>] | nominal order and spacing of motifs is specified by <diag> which is a block diagram |
| DNA-Only Options | |
| [-norc] | do not score reverse complement DNA strand |
| [-sep] | score reverse complement DNA strand as a separate sequence |
| [-dna] | translate DNA sequences to protein; motifs must be protein; sequences must be DNA |
| [-comp] | adjust *p*-values and *E*-values for sequence composition |
| Which Results To Print | |
| [-ev <ev>] | print results for sequences with *E*-value < <ev> (default: 10) |
| Appearance Of Block Diagrams | |
| [-mt <mt>] | show motif matches with *p*-value < <mt> (default: 0.0001) |
| [-w] | show weak matches (<mt> < *p*-value < <mt>\*10) in angle brackets in the hit list or when the xml is converted to text |
| [-best] | include only the best motif hits in -hit\_list diagrams |
| [-seqp] | use SEQUENCE *p*-values for motif thresholds (default: use POSITION *p*-values) |
| Miscellaneous | |
| [-mf <mf>] | in results use <mf> as motif file name |
| [-df <df>] | in results use <df> as database name (ignored when -dblist) |
| [-dl <dl>] | in results use <dl> as link to search sequence names; token SEQUENCEID is replaced with the FASTA sequence ID; ignored when -dblist; |
| [-minseqs <ms>] | lower bound on number of sequences in db |
| [-nostatus] | do not print progress report |
| [-notext] | do not create text output |
| [-nohtml] | do not create html output |

MAST is a tool for searching biological sequence databases for sequences
that contain one or more of a group of known motifs.

A motif is a sequence pattern that occurs repeatedly in a group of related
protein or DNA sequences. Motifs are represented as position-dependent
scoring matrices that describe the score of each possible letter at each
position in the pattern. Individual motifs may not contain gaps. Patterns with
variable-length gaps must be split into two or more separate motifs before
being submitted as input to MAST.

MAST takes as input a file containing the descriptions of one or more motifs
and searches a sequence database that you select for sequences that match
the motifs. The motif file can be the output of the MEME motif discovery tool
or any file in the appropriate format.

MAST outputs an xml file which can then be converted into html or text format.
The xml file is designed for machine processing and the html file is designed
for human viewing. The text format is available for backwards compatibility
though due to design decisions made to optimise the xml for html generation
the output for separate scoring mode is not identical and some options were
removed. The text format will be unsupported in future releases and so we
recommend you migrate any programs reading mast output to the xml format.

MAST outputs three things:

1. The names of the high-scoring sequences sorted by the strength of the
   combined match of the sequence to all of the motifs in the group.
2. Motif diagrams showing the order and spacing of the motifs within each
   matching sequence.
3. Detailed annotation of each matching sequence showing the sequence
   and the locations and strengths of matches to the motifs.

MAST works by calculating match scores for each sequence in the database
compared with each of the motifs in the group of motifs you provide. For each
sequence, the match scores are converted into various types of *p*-values and
these are used to determine the overall match of the sequence to the group of
motifs and the probable order and spacing of occurrences of the motifs in the
sequence.

MAST generates a human readable file from the xml output containing:

- the version of MAST and the date it was built,
- the reference to cite if you use MAST in your research,
- a description of the databases and motifs used in the search,
- an explanation of the result,
- the sequences identifier and score sorted by score matching the group of motifs
  above a stated level of statistical significance,
- motif diagrams showing the order and spacing of occurrences of the
  motifs in the significant sequences and,
- annotated sequences showing the positions and *p*-values of all motif
  occurrences in each of the high-scoring sequences.

The html version is the recommended version for human reading and has all
sections documented however the text version has no documentation for the first section.
That section lists each motif along with the sequence that would achieve the
best possible match score. In order to avoid biased scores when multiple motif
scores are combined, MAST also computes the pairwise correlations between
each pair of motifs. The correlation between two motifs is the maximum sum of
Pearson's correlation coefficients for aligned columns divided
by the width of the shorter motif. The maximum is found by trying all alignments
of the two motifs. Motifs with correlations below 0.60 have little effect
on the accuracy of the combined scores. Pairs of motifs with higher correlations
should be removed from the query.

## Match Scores

The match score of a motif to a position in a sequence is the sum of the
score from each column of the position-dependent scoring matrix
corresponding to the letter at that position in the sequence. For example, if
the sequence is

```
       TAATGTTGGTGCTGGTTTTTGTGGCATCGGGCGAGAATAGCGC
          ========
```

and the motif is represented by the position-dependent scoring matrix (where
each row of the matrix corresponds to a position in the motif)

| Position | A C | G | T | |  |  |  |
| --- | --- | --- | --- | --- | --- | --- | --- |
| 1 | 1.447 | 0.188 | -4.025 | -4.095 |
| 2 | 0.739 | 1.339 | -3.945 | -2.325 |
| 3 | 1.764 | -3.562 | -4.197 | -3.895 |
| 4 | 1.574 | -3.784 | -1.594 | -1.994 |
| 5 | 1.602 | -3.935 | -4.054 | -1.370 |
| 6 | 0.797 | -3.647 | -0.814 | 0.215 |
| 7 | -1.280 | 1.873 | -0.607 | -1.993 |
| 8 | -3.076 | 1.035 | 1.414 | -3.913 |

then the match score of the fourth position in the sequence (underlined)
would be found by summing the score for T in position 1, G in position 2 and
so on until G in position 8. So the match score would be

```
         score = -4.095 + -3.945 + -3.895 + -1.994
                 + -4.054 + -0.814 + -1.933 + 1.414 
               = -19.316
```

The match scores for other positions in the sequence are calculated in the
same way. Match scores are only calculated if the match completely fits within
the sequence. Match scores are not calculated if the motif would overhang
either end of the sequence.

## *P*-values

MAST reports all matches of a sequence to a motif or group of motifs in terms
of the *p*-value of the match. MAST considers the *p*-values of four types of
events:

- position *p*-value: the match of a single position within a sequence to
  a given motif,
- sequence *p*-value: the best match of any position within a sequence
  to a given motif,
- combined *p*-value: the combined best matches of a sequence to a
  group of motifs, and
- *E*-value: observing a combined *p*-value at least as small in a random
  database of the same size.

All *p*-values are based on a random sequence model that assumes each
position in a random sequence is generated according to the average letter
frequencies of all sequences in the appropriate (peptide or nucleotide)
non-redundant database (ftp://ncbi.nlm.nih.gov/blast/db/) on September 22,
1996. This can be overridden by specifying the `-bfile`
or `-comp` options (see below). For DNA sequences,
unless `-norc` is given, the positive and reverse
complement strand frequencies are averaged together.

1. `-bfile <bfile>`
   The random model uses the letter frequencies given in
   `<bfile>` instead of the non-redundant database frequencies.
   The format of `<bfile>` is the same as that for the MEME
   `-bfile` option; see the MEME documentation for details.
   You can create files in the appropriate format based on the base/residue
   composition of your own FASTA sequence files using the
   command "fasta-get-markov" included in the MEME distribution. Type
   `fasta-get-markov` on the command line for documentation.
   (Sample files are also given in
   directory tests: `tests/nt.freq` and `tests/na.freq`.)
2. `-comp`
   The random model uses the letter frequencies in the current target
   sequence instead of the non-redundant database frequencies. This
   causes *p*-values and *E*-values to be compensated individually
   for the actual composition of each sequence in the database. This option
   can increase search time substantially due to the need to compute
   a different score distribution for each high-scoring sequence.
   With this option and DNA sequences, the positive and reverse complement
   strand frequencies are *not* averaged together.

### Position *p*-value

The *p*-value of a match of a given position within a sequence to a
motif is defined as the probability of a randomly selected position in a
randomly generated sequence having a match score at least as large
as that of the given position.
**Note:**If MAST is combining reverse complement DNA strands,
the position *p*-value is **not** corrected for multiple tests.

### Sequence *p*-value

The *p*-value of a match of a sequence to a motif is defined as the
probability of a randomly generated sequence of the same length
having a match score at least as large as the largest match score of
any position in the sequence.

### Combined *p*-value

The *p*-value of a match of a sequence to a group of motifs is defined
as the probability of a randomly generated sequence of the same
length having sequence *p*-values whose product is at least as small
as the product of the sequence *p*-values of the matches of the motifs
to the given sequence.

### *E*-value

The *E*-value of the match of a sequence in a database to a a group
of motifs is defined as the expected number of sequences in a random
database of the same size that would match the motifs as well as the
sequence does and is equal to the combined *p*-value of the sequence
times the number of sequences in the database.

## High-scoring Sequences

MAST lists the names and part of the descriptive text of all sequences
whose *E*-value is less than *E*. Sequences shorter than one or more of the
motifs are skipped. The sequences are sorted by increasing *E*-value. The
value of *E* is set to 10 for the WEB server but is user-selectable in the
down-loadable version of MAST.

## Motif Diagrams

Motif diagrams show the order and spacing of non-overlapping matches to
the motifs in each high-scoring sequence. Motif occurrences are determined
based on the position *p*-value of matches to the motif. Strong matches
(*p*-value < *M*) are shown in square brackets (`[ ]'), weak matches (*M* <
*p*-value < *M* x 10) are shown in angle brackets (`< >') and the length of
non-motif sequence ("spacer") is shown between underscores (`\_'). For example,

```
             27_[3]_44_<4>_99_[1]_7
```

shows an initial spacer of length 27, followed by a strong match to motif 3, a
spacer of length 44, a weak match to motif 4, a spacer of length 99, a strong
match to motif 1 and a final non-motif sequence of length 7. The value of *M* is
0.0001 for the WEB server but is user-selectable in the downloadable
version of MAST.

## Annotated Sequences

MAST annotates each high-scoring sequence by printing the sequence
along with the position and strength of all the non-overlapping motif
occurrences. The four lines above each motif occurrence contain,
respectively,

- the motif number of the occurrence,
- the position *p*-value of the occurrence,
- the best possible match to the motif, and
- a plus sign (`+') above each letter in the occurrence that has a positive
  match score to the motif.

The best possible match to a motif is the sequence of letters which would
achieve the highest match score.

## Hit List

If you specify the `-hit_list` switch to MAST, MAST outputs ONLY a
list of "hits" in easily machine-readable format.
Each line corresponds to one motif occurrence in one sequence.
The format of the hit lines is

```
        [<sequence_name> <strand><motif> <start> <end> <score> <p-value>]+
```

where

```
        <sequence_name> is the name of the sequence containing the hit
        <strand>        is the strand (+ or - for DNA, blank for protein),
        <motif>         is the motif number,
        <start>         is the starting position of the hit,
        <end>           is the ending position of the hit, and
        <score>         is the score the hit,
        <p-value>       is the position p-value of the hit.
```

Two comment lines (starting with "#") are written above the list of hits,
and the MAST command line is printed as a comment line after the list.
An example of the output using the `-hit_list` switch to MAST is:

```
        # All non-overlapping hits in all sequences.
        # sequence_name motif hit_start hit_end score hit_p-value
        ce1cg -2 8 22  1459.90 1.67e-06
        ara +2 2 16  1661.18 5.04e-08
        bglr1 +2 1 15  1274.97 1.42e-05
        cya -2 19 33  1101.37 6.64e-05
        gale +2 5 19  1076.21 8.11e-05
        ilv -2 6 20  1098.85 6.78e-05
        malk +2 37 51  1085.02 7.56e-05
        ompa +2 5 19  1583.18 2.43e-07
        # mast tests/meme/meme.crp0.oops tests/common/crp0.s -hit_list -m 2
```

## Loading Multiple Sequence Databases

Multiple sequence databases can be loaded by MAST by putting the file names
into a file and specifying that file instead of the sequence database with the
option -dblist.

The file list has one file name on each line with the optional name and link
as follows:

```
      <file> [<name> <link>]
      ...
      ...
```

If it is specified then the name will be used instead of the file name in
the output. If the link is specified then all sequences for that database in
the html output will have a hyperlink to the URL specified with the text
SEQUENCEID replaced with the FASTA sequence id.

## EXAMPLES:

The following examples assume that file "meme.results" is the
output of a MEME run containing at least 3 motifs which was
created on the trainingset "training.fasta" and file
SwissProt is a copy of the Swiss-Prot database on your local disk.
DNA\_DB is a copy of a DNA database on your local disk.

1. Annotate the training set:

   ```
           mast meme.results training.fasta
   ```
2. Find sequences matching the motif and annotate them in
   the SwissProt database:

   ```
           mast meme.results SwissProt
   ```
3. Show sequences with weaker combined matches to motifs.

   ```
           mast meme.results SwissProt -ev 200
   ```
4. Include a nominal order and spacing of the first three motifs
   in the calculation of the sequence *p*-values to increase the
   sensitivity of the search for matching sequences:

   ```
           mast meme.results SwissProt -diag "9-[2]-61-[1]-62-[3]-91"
   ```
5. Use only the first and third motifs in the search:

   ```
         mast meme.results SwissProt -m 1 -m 3
   ```
6. Use only the first two motifs in the search:

   ```
           mast meme.results SwissProt -c 2
   ```
7. Search DNA sequences using protein motifs, adjusting *p*-values and *E*-values
   for each sequence by that sequence's composition:

   ```
           mast meme.results DNA_DB -dna -comp
   ```
